# Categorization of tinnitus in view of history and medical discourse

**DOI:** 10.3402/qhw.v8i0.23530

**Published:** 2013-12-20

**Authors:** Soly Erlandsson, Nicholas Dauman

**Affiliations:** 1 Department of Social and Behavioural Studies, University West, Sweden; 2 Department of Psychology, University of Poitiers, France

The foremost, dominant, and influential scientific discourse of how to define tinnitus states that tinnitus is *the perception of sound(s) in the absence of an external sound source*. This is the most common statement among researchers in audiology and related fields, stemming from basic neurosciences (Kaltenbach, 2011) to applied psychophysiology (Kropp et al., 2012), audiology (Caffier et al., 2006), and behavioural psychology (Westin et al., 2008). It is puzzling that scientific affiliation and paradigms have had no influence on how the condition is defined as for instance one would expect psychologists and neurologists to have their own viewpoint on this issue. The current definition of tinnitus has an old tradition in the history of audiology. Exactly two centuries ago, the French otologist Jean Marc Gaspard Itard (1812) distinguished two basic forms of tinnitus just as the neuroscientist Jos Eggermont (2012) currently characterized tinnitus. Between those two historical references, a number of other researchers within the field have identically labelled two forms of tinnitus, depending on the fashionable terms of their time. Itard and Eggermont, respectively, label one form of tinnitus that is very rare as “true” (Itard) and “objective” (Eggermont) tinnitus. These are sounds that are perceived in the ear *by the patient and* *the* *clinician*, when the latter is close enough to the sound's source in the body of the patient or can use an amplifier to hear it. Most of the time, however, tinnitus is a phenomenon, which cannot be heard by anyone else *other than the patient him- or herself*. The only material that the clinician can refer to is the patient's testimony of what is perceived, with loudness sometimes hardly sustainable. Itard (1812) and Eggermont (2012), respectively, label this second form of tinnitus “false” and “subjective” tinnitus, that is, when no physical stimulation arises from the body of the perceiver. Notably, this distinction was already made three centuries ago, in the same terms, by Joseph Guichard Duverney (1683, p. 161): “true tinnitus” being “the perception of a sound that is internal” and “false tinnitus” being “a sound that is not.” Contrary to basic advances in the field of hearing impairment, major technical discoveries in audiology have brought no fundamental changes to this common statement. A number of quotes can be raised with reference to the reasons that are behind the current research position.

The standard definition of tinnitus has its roots in a medical discourse, and a regime that has the power to classify what tinnitus is and what tinnitus is not. Power can be useful and can contribute to the discourse as the force behind the struggle to find ways in which an alternative statement of meaning of how to apprehend tinnitus can be introduced. We hypothesize that the present “common sense” definition of tinnitus, a definition that has reached the status of being the true concept, will not be abandoned or supplemented unless a new discourse favours research within the same paradigm. Or, possibly if someone high in hierarchy comes up with a definition that can be accepted by those who proclaim their belonging to this paradigm. It is appealing to notice that the use of “a sound” does not seem to have any bonds to the body, otherwise common in medical discourse where the body is the nodal point around which other connotations are materialized (Jörgensen & Phillips, 2012). You may argue that the ear is a body organ, but sounds reaching the ear canal are external, which is precisely what is claimed by the common definition of tinnitus. Impressions, excitements, feelings, emotions, tensions, or sensations which can arise within the body may or may not be decoded and turned into something else, for example a sound or noise. By turning the focus on a source being outside the body, the subjective experience that the patient describes becomes objectified and devalued. Perhaps the choice of “a sound” can be explained by its linkage to a process, under which fixation of meaning has become so customary (habitual) that it, in a sense, seems entirely logical to those who are involved.

Tinnitus exists independently of a medical classification system, but whether or not it is labelled an “inner sound without any external sound source” or seen as “the manifestation of existential anxiety” depends on the discursive context in which it occurs. Hypothetically, according to the theory by Laclau and Mouffe (1985), the latter example would belong to the field of discursivity and is placed outside the dominating discourse, that is, a conception that would be excluded by the discourse in charge. There are social consequences of how we refer to and label a phenomenon. A particular discourse has an influence on the way we act, our search for knowledge, and how we meet the consulting patient, who might learn that “tinnitus is the perception of a sound in the absence of an external sound source.” Moreover, as pointed out by Jörgensen and Phillips (2012, p. 13) in view of Foucault's theses (Foucault, 1972), “The historical rules of the particular discourse delimit what it is possible to say.” In short, it limits our possibilities for action. Hegemony, in discourse analytical terms, indicates that there is a dominance of one particular perspective. In science, the most productive circumstance would be one in which several discourses can exist side by side, an insight worth considering by those who are active within the field of tinnitus research.

The consequences of defending the present “one-sided” definition of tinnitus are also likely to be found on how rehabilitation is organized. A complication arises when tinnitus is compared to an external sound, because it leans explicitly on an analogy between tinnitus and the experience of hearing (Dauman, Erlandsson, & Carlsson, 2013). By conceptualizing tinnitus as a sound, there is a risk that the consulting dialogue will focus more on the physical characteristics of tinnitus (a sound) than on the patients’ own comprehension of what the condition has implied for their psychological health and life. It is not uncommon that the clinicians underestimate the impact their own strongly held beliefs has on their clients and how this governs what is being said during the consultation. The fact that no epidemiological data so far have demonstrated that the populations of tinnitus patients in any way are homogeneous, the definition of tinnitus as the perception of a sound, becomes even more incomprehensible. How then can we describe what tinnitus is? Generally, clients believe that understanding what it means to have tinnitus implies being someone who shares this experience with them. To begin with, consulting the patients and collecting their images of what tinnitus is can be an alternative to the professionals’ standardized description. Another alternative would be to start from each discipline having an interest in the research field and search for conceptual models that can be applied in research on tinnitus. In this way, new and somewhat progressive concepts may find their way from the field of discursivity to the current discourse on tinnitus.

*Soly Erlandsson* Department of Social and Behavioural Studies University West Sweden*Nicholas Dauman* Department of Psychology University of Poitiers France
